# Probing the Electrode–Electrolyte Interface of Sodium/Glyme-Based Battery Electrolytes

**DOI:** 10.1021/acs.jpcc.3c08083

**Published:** 2024-03-27

**Authors:** Dodangodage Ishara Senadheera, Orlando Carrillo-Bohorquez, Ernest O. Nachaki, Ryan Jorn, Daniel G. Kuroda, Revati Kumar

**Affiliations:** † Department of Chemistry, 232 Choppin Hall, Louisiana State University, Baton Rouge, Louisiana 70803, United States; ‡ Department of Chemistry, Villanova University, Villanova, Pennsylvania 19085, United States

## Abstract

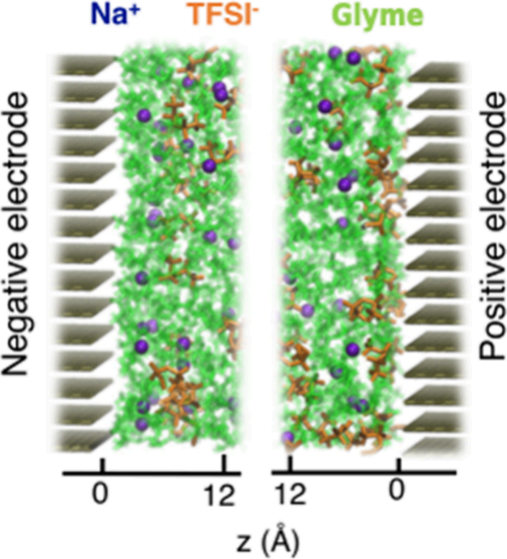

Sodium-ion batteries (NIBs) are promising systems for large-scale energy storage solutions; yet, further enhancements are required for their commercial viability. Improving the electrochemical performance of NIBs goes beyond the chemical description of the electrolyte and electrode materials as it requires a comprehensive understanding of the underlying mechanisms that govern the interface between electrodes and electrolytes. In particular, the decomposition reactions occurring at these interfaces lead to the formation of surface films. Previous work has revealed that the solvation structure of cations in the electrolyte has a significant influence on the formation and properties of these surface films. Here, an experimentally validated molecular dynamics study is performed on a 1 M NaTFSI salt in glymes of different lengths placed between two graphite electrodes having a constant bias potential. The focus of this study is on describing the solvation environment around the sodium ions at the electrode–electrolyte interface as a function of glyme chain length and applied potential. The results of the study show that the diglyme/TFSI system presents features at the interface that significantly differ from those of the triglyme/TFSI and tetraglyme/TFSI systems. These computational predictions are successfully corroborated by the experimentally measured capacitance of these systems. In addition, the dominant solvation structures at the interface explain the electrochemical stability of the system as they are consistent with cyclic voltammetry characterization.

## Introduction

Global energy demand^1^ underscores the need to explore and expand efficient pathways of generating and storing power.^1−3^ Among the various energy storage technologies, electrochemical energy storage stands out as an appealing choice due to its high energy conversion efficiency, compact size, and rapid response capabilities.^4^ Electrochemical devices, like lithium-ion batteries (LIBs), have taken a prominent position in both the electric vehicle and personal electronics industries due to their notable reversibility and energy density.^5^ However, the cost of materials containing lithium may hinder the availability of lithium-ion technologies for large-scale energy storage systems^6,7^ and consequently their extensive application.^8^ Conversely, sodium, accounting for 2.8% of the Earth’s crust, combines greater abundance with physical and chemical resemblance to lithium, making sodium-ion batteries (NIBs) a promising replacement in the realm of beyond-lithium technology.^9−11^

Electrolytes, such as lithium salts solvated in ethylene carbonate, propylene carbonate, and ethyl methyl carbonate, have been investigated and fine-tuned to enhance the performance of LIBs.^12^ However, these carbonate-based solvents present safety issues arising from high volatility and flammability.^13^ On the other hand, ether solvents have recently gained greater emphasis in sodium ion and sodium air systems as an alternative to carbonates.^14−16^ Ether solvents belonging to the glyme series have a structural composition represented as repeating units of CH_3_–(O–CH_2_)_*n*_–O–CH_3,_^17,18^ with the most popular being the first four in the series: monoglyme, diglyme, triglyme, and tetraglyme, corresponding to *n* = 1, 2, 3, and 4, respectively. These ether-based solvents possess unique solvating power derived from chelating effects. Chelation replaces multiple monodentate ligands (such as carbonates in conventional battery electrolytes) coordinating with the alkali metal ion with a single moiety (in this case, glyme), which is entropically favored. This translates into higher coordination numbers compared to typical carbonate solvents.^19,20^ Previous studies have shown that glyme-based solvents with sodium salts have two glyme molecules complexing with the cation in the case of diglyme and triglyme systems, and one to two distinct glyme molecules in the solvation shells of tetraglyme systems.^21^ The chelating effect is particularly pronounced in longer glymes resulting in increased oxidative stability.^16,22,23^ Electronic structure calculations show that the interactions between metal cations and the oxygen atoms of glymes lead to a notable reduction in the highest occupied molecular orbital (HOMO) energy levels of glymes.^16,22^ Furthermore, glymes also have the excellent property of cointercalation with Na ions into graphite, directly participating in the sodium ion storage process.^24−26^ Regarding electrode materials, carbon-based substances like hard carbon and expanded-graphite exhibit promise to serve as anodes in NIBs because of the cost-effectiveness of carbonaceous materials and suitable charge/discharge plateau.^27−31^

Improving the electrochemical performance of NIBs requires more than just a knowledge of electrolytes and electrodes. Hence a thorough comprehension of the molecular processes governing the interface formation between electrodes and electrolytes is needed. Compared with bulk, intricacies due to the disruption of the dielectric constant across the interface demand a deeper characterization of the distribution and dynamics of the charged species in these regions. The permeation of the cations (in both NIBs and LIBs) from the electrolyte to the electrode is a slow process compared to the migration of cations through the electrolyte during charging/discharging. However, this slow process determines the battery performance.^32^ In addition, the unavoidable reduction and oxidation of electrolyte components near the solid–liquid interface further complicate this process.^33^ When the electrolyte comes into contact with the anode, it tends to undergo reduction, resulting in the creation of the solid electrolyte interphase (SEI) on the anodic surfaces. Similarly, organic electrolytes prone to oxidation, lead to the formation of surface films referred to as the cathode–electrolyte interphase (CEI).^34,35^ Prior investigations have revealed that the solvation patterns and networks of cations within the electrolyte substantially impact the development and characteristics of the SEI/CEI on the electrode surface.^36,37^ It is essential to conduct systematic investigations of electrolyte behavior near the electrode–electrolyte interface in NIBs to gain further insights into the coupled nature of electrolyte composition to subsequent film-forming side reactions.

Molecular dynamics studies of the electrode/electrolyte interface have employed different approaches to account for the applied bias voltage and electrode polarization by the solvated ions, including the image charge method and the constant potential method (CPM).^38−43^ Jorn and co-workers^39^ performed molecular dynamics using an image charge method to model and study the electrode–electrolyte interface in Li-ion batteries consisting of a relatively thin SEI. Electrode/electrolyte studies of mixed carbonate/LiPF_6_ electrolytes near clean graphite surfaces^44^ and sulfolane-based electrolytes at graphitic electrodes^38^ have also been studied. Regarding glyme-based electrolytes, interfacial studies of LiTFSI solvated in tetraglyme using the CPM have been reported.^40^ However, there is a relatively limited amount of research directed toward investigating the electrode–electrolyte interface of sodium/glyme-based systems.

The interaction of glymes with sodium cations in bulk electrolytes has been considered in previous work through a blend of computational and spectroscopic approaches.^17,45−47^ Most of these investigations made use of accurate yet computationally efficient force fields to study the proportions of solvent molecules and anions within cation solvation shells and characterize their association with anions (e.g., free ions, contact ion pairs, solvent-separated ion pairs, and aggregates). Expanding upon these previous sodium/glyme studies, this research pivots to the chemistry taking place at the formation of interfaces between graphite electrodes and electrolytes of NaTFSI (sodium bis(trifluoromethylsulfonyl)imide) dissolved in glyme solvents at different applied potentials. The present work aims to understand the solvation structure of sodium cations at the electrode–electrolyte interface with specific attention paid to the impact of glyme chain length and applied voltage. Specifically, the solvation structures at the interface are compared with the bulk solvation structures for each electrolyte. This approach provides us with the ability to examine the variations in solvation environments at polarized surfaces in relation to the bulk, shedding potentially new light on the initial steps of electrolyte degradation in ether/NaTFSI electrolytes and ion transport.

The article is arranged as follows. In the following section, the methodology used to study three different glyme systems with MD simulations (that includes NaTFSI in di-, tri-, and tetraglyme), in bulk liquid and at the electrode interface, is outlined. In addition, a description of the method used for HOMO–LUMO energy gap calculations is presented as well as experimental details on sample preparation and the linear sweep and cyclic voltammetry techniques. The subsequent section ties together the experimental measurements with the combination of classical simulations and DFT calculations: the calculated Poisson potentials, potential of zero charge (PZC), density profiles, solvation structures, and HOMO–LUMO energies. Finally, this article summarizes the work and highlights the remarkable differences found for diglyme/NaTFSI in comparison with the other electrolytes.

## Computational Methodology

**Scheme 1:**
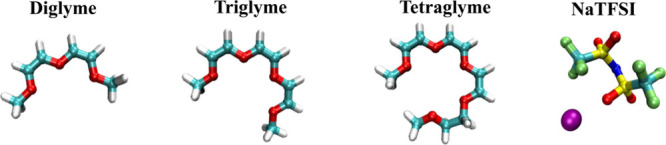
Chemical Structures of Diglyme, Triglyme, Tetraglyme, and NaTFSI Molecules Red, white, cyan, purple, blue, yellow, and green colors represent oxygen, hydrogen, carbon, sodium, nitrogen, sulfur, and fluorine atoms, respectively.

Systems of Na^+^ and TFSI^–^ ions solvated in glymes of different lengths, including diglyme, triglyme, and tetraglyme, were considered (structures are represented in Scheme 1). Initially, bulk MD simulations were carried out with the LAMMPS software package,^48^ using a simulation box with side lengths of 50 Å × 50 Å × 100 Å. Initial configurations were set up randomly with Packmol.^49^ A total of 150 [Na^+^]/[TFSI^–^] ions were considered in every system to achieve a concentration of 1 M. The numbers of glyme molecules employed in each simulation are presented in Table S1. Periodic boundary conditions in all directions were employed, and long-range electrostatic interactions were treated using the particle–particle–particle–mesh (PPPM) method. The cutoff radius was set to 6 Å for both the LJ and real space Coulombic interactions. The modified class II Polymer Consistent Force Field (PCFF), which includes parametrized values for ion–ion and ion–solvent interactions specific to NaTFSI/glyme systems established in our prior research,^46^ was implemented in this study and all the simulations were carried out with a time step of 1 fs.

The bulk diglyme/NaTFSI system was minimized and equilibrated at 300 K in the NVT, the NPT (box dimensions were allowed to vary in all three directions), and finally the NVE ensembles for 5 ns each (for a total of 15 ns) followed by a 40 ns production run in the NVE ensemble. For bulk simulations of triglyme/NaTFSI and tetraglyme/NaTFSI, two simulations for each system were conducted, starting with two different configurations to achieve better sampling and to avoid the consequences attributed to the slow dynamics inherent in large molecules. Initially, the systems were minimized and then linearly heated from 0 to 320 K over 5 ns followed by equilibration runs of 5 ns each in the NVT and NPT ensembles at 320 K, without constraining any of the dimensions for the latter case. The second configuration for each system was obtained by linearly heating the equilibrated system at 320 to 340 K over 5 ns and then slowly cooling back to 320 K at a rate of 1 K/ns. Finally, a production run of 20 ns each in the NVE ensemble was performed for each triglyme and tetraglyme systems.

**Figure 1:**
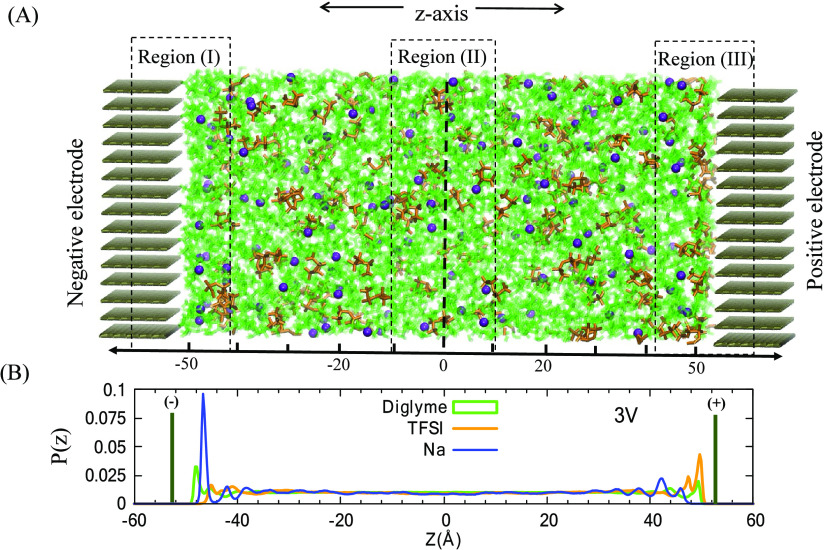
(a) A snapshot of the electrode/electrolyte simulation cell extracted from the diglyme/NaTFSI system at 3 V. The graphite electrodes are represented in tan shades, while diglymes, sodium cations, and TFSI anions are visualized respectively in green, purple, and orange shades. (b) Normalized probability density distribution *P*(*z*) (normalized to have unit area) for each species of the above system as a function of the position *z* (diglyme, TFSI^–^, and Na^+^ are represented in green, orange, and blue lines, respectively). The positions of the electrode surfaces are depicted by vertical lines.

Simulations of a capacitor model were performed with the ELECTRODE package,^50^ implemented in LAMMPS. Graphite electrodes were modeled as 15 stacked graphene sheets perpendicular to the *z* direction of the simulation box, as shown in Figure 1a, and treated as ideal conductors. This *z* direction was considered as the direction of the electric field due to the voltage applied over the electrodes and consequently regarded as the nonperiodic direction. The graphite cell measurements are approximately 50 Å × 50 Å × 130 Å featuring electrodes (having dimensions of around 50 Å × 50 Å × 12.3 Å) placed at opposing ends with the electrolyte filling the intervening space (Figure 1a). The electrode atoms were fixed during simulations and the interelectrode separation was 105 Å. The box length in the *z* direction was chosen based on previous work so as to minimize the interaction between the electrodes,^39,41^ thereby ensuring a plateau in the Poisson potential in the bulk-like region when a voltage is applied, while maintaining a computationally tractable simulation box.

Final equilibrated configurations for the bulk simulations described above were used as starting points for the electrode simulations. The final numbers of glyme molecules and the amount of [Na^+^]/[TFSI^–^] ions required to ensure the 1 M concentration in the new cells are given in Table S1, along with the final dimensions for each of the simulation boxes.

The fix electrode/conp style implemented in the ELECTRODE package of LAMMPS (2023 v.) was employed to simulate an applied voltage across the cell. This fix style implements a constant potential method (CPM), and each system was studied at three different applied voltages: 0 V, 1 V, and 3 V. The constructed cell for diglyme/NaTFSI was equilibrated for 10 ns followed by a production run of 40 ns in the NVT ensemble at 300 K. The four cells (two each) constructed for triglyme/NaTFSI and tetraglyme/NaTFSI systems were equilibrated for 20 ns and then followed by production runs of 20 ns each in the NVT ensemble at 320 K (40 ns in total for production runs for each system). The final trajectories in each case were used to calculate the Poisson potential across the cells, normalized probability density distributions of each species along the *z*-direction, and to study sodium ion solvation environments at interfacial regions as a function of the applied voltage difference and glyme chain length.

Comparison with experimental voltammetry was accomplished by considering the molecular orbitals of the most populated solvation structures. The HOMO–LUMO gap as a function of glyme chain lengths was calculated using the PBE functional along with the 6–31+G(d,p) basis set in Gaussian 16.^51^ For each electrolyte, the most probable solvation structures were taken from the MD trajectories as the geometries for the electronic structure calculations. The level of theory and basis set were selected based on previously demonstrated accuracy in describing sodium-ion electrolytes.^52^

## Experimental Methodology

### Sample Preparation

Sodium bis(trifluoromethanesulfonyl)imide (NaTFSI, 99.95% AmBeed), bis(2-methoxyethyl) ether (CH_3_(OCH_2_CH_2_)_2_OCH_3_, diglyme ≥ 99.0% Acros Organics), triethylene glycol dimethyl ether (CH_3_(OCH_2_CH_2_)_3_OCH_3_, triglyme ≥ 99.5% Millipore Sigma), tetraethylene glycol dimethyl ether (CH_3_(OCH_2_CH_2_)_4_OCH_3_, tetraglyme 99.0% Acros Organics), and ferrocene (Fc, 99% Beantown Chemical) were used without further purification. NaTFSI was dried in a vacuum oven at a temperature of 120 °C for 24 h. Diglyme (G2), triglyme (G3), and tetraglyme (G4) were dried in 4 Å molecular sieves for at least 48 h before use. Solutions of NaTFSI (1.2 M) were prepared by dissolving NaTFSI in the respective glymes (G1, G2, and G3) in a nitrogen filled glovebox.

### Electrochemical Measurements

Linear sweep (LSV) and cyclic voltammetry (CV) measurements were performed using a WaveDriver 100 Potentiostat (Pine Research) and a ceramic Platinum Screen Printed Electrode (Pt-SPE, RRPE2011PT-6 Pine Research) consisting of a 2 mm Platinum Working Electrode (Pt-WE), a Platinum Counter Electrode (Pt-CE), and a Ag pseudoreference (Ag/Ag+) electrode. All LSV and CV measurements were performed with respect to ferrocene as an internal standard. The NaTFSI glyme solutions were purged with nitrogen for at least 45 min before the LSV measurements. The electrochemical cell was allowed to equilibrate for at least 30 min to stabilize the Ag/Ag+ pseudoreference electrode potential. Electrochemical measurements of the electrolytes were performed in a nitrogen-filled glovebox with positive pressure at 25 °C. The capacitance was determined from the area of the cyclic voltammogram using the formulation previously presented by Li et al.^53^

## Results and Discussion

### Simulations of Electrode/Electrolyte Interface

The molecular dynamics study simulated three systems of 1 M NaTFSI/glyme-based electrolytes, comprising diglyme, triglyme, and tetraglyme under three applied voltages (0, 1, and 3 V) across the positively and negatively charged electrode surfaces. Note that these nonreactive electrode/electrolyte models resemble a capacitor model rather than a functioning battery where redox reactions take place.

The analyses were performed over the entire production length of each of the MD simulations. Figure 1a presents an “equilibrated” snapshot from the simulation of diglyme/NaTFSI at the applied voltage difference of 3 V across the cell, where the negative potential (−1.5 V, negative electrode) is on the left, and the positive potential (+1.5 V, positive electrode) is on the right. The asymmetric distribution of the three components at the negative and positive interfacial regions can be visualized through the normalized probability density distributions, *P(z)*, along the *z*-axis of the cell for oxygens atoms of diglyme, TFSI anions, and sodium cations comprising the electrolyte (Figure 1b). A comparison of these density distributions for all three glyme/NaTFSI-based systems as a function of applied voltage differences, 0, 1, and 3 V, is provided in the Supporting Information (SI), in Figures S1a–c (distributions across the entire cell), and in Figures S1d–f (distributions near the electrode surfaces). When examining density profiles at 0 V for all three glymes, it is possible to observe that the first interfacial layer starts around 1.6 Å from the electrodes, and bulk densities are not recovered until about 15 Å from either electrode (electrodes are placed at −52.5 Å and + 52.5 Å). A significant observation is that, at the potential differences considered, the sodium ions (indicated by the blue lines) do not approach the electrode surfaces, unlike the TFSI anions and glyme molecules. This indicates that, even at the interfacial regions, sodium ions prefer to remain solvated by glyme molecules and the chelation effect dominates over the attractive interaction with the charged surface. On the other hand, TFSI exhibits a tendency to closely approach the electrode surface at 0 V as well as the positive electrode at nonzero potential differences. To provide a connection with electrochemical measurements, the calculated distributions of species at the interfaces are considered through the Poisson potential.

### Poisson Potential and Potential of Zero Charge (PZC)

,The electrostatic potential across the simulation cell as a function of *z* (Å) was obtained by integrating the 1-D Poisson equation:
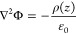
where ϕ(*z*) is the potential difference across the cell, ρ(*z*) is the equilibrium distribution of charge density, and ε_0_ is the vacuum permittivity. The Poisson potentials across the cell calculated for the three applied voltages (0, 1, and 3 V) on the diglyme/NaTFSI electrolyte are presented in Figure 3a. Notably, Poisson profiles for triglyme/NaTFSI and tetraglyme/NaTFSI are very similar (see Figures S2a and b in the SI). In all cases, the calculated potential differences across the simulation cell matches the applied voltage, validating the CPM used in this study. Note that this calculation assumes that all charges contribute equally to the Poisson profile. However, a delocalization effect and charge transfer in and around the ionic species can be correctly described only using *ab initio* methods. Hence, the contribution of charge arising from ion pairing in these classical MD simulations is likely to be overestimated, and consequently, the PZC values as well.

The oscillation in the Poisson profiles close to either interface in Figure 3a provides evidence of the formation of electric double layers (EDLs). Moreover, the EDL formation becomes clearer as the applied voltage increases because of the accumulation of ordered ions at the electrode/electrolyte interface (see Figure S1d–f, SI). Interestingly, the peak positions of the double layer in each glyme system remains constant across different voltages, but a variation in peak heights is observed. Specifically, diglyme exhibits the lowest peak height of the three. Triglyme and tetraglyme peaks display similar heights (Figure S2c, in the SI, presents a zoomed-in view of the Poisson potentials at the interfaces). This result suggests that the diglyme system is markedly different from that of the longer glymes.

**Figure 2:**
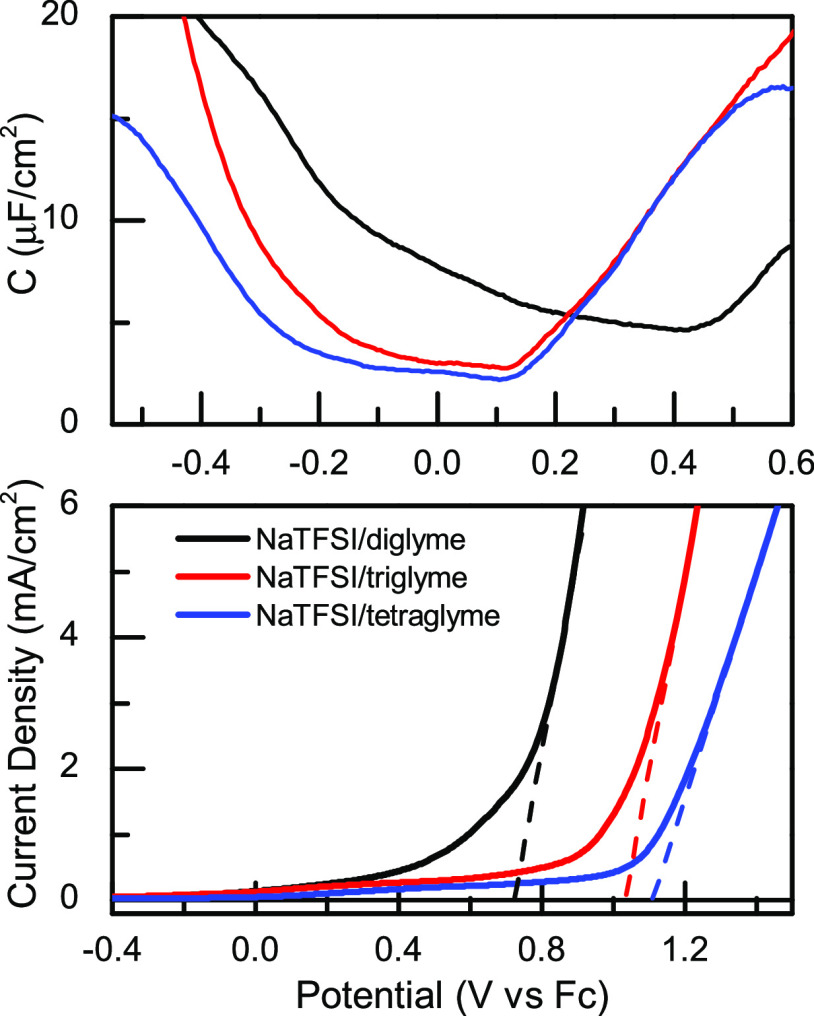
Capacitance and electrochemical window of 1 M NaTFSI in G2 (black curve), G3 (red curve), and G4 (blue curve). Top panel contains the capacitances determined from cyclic voltammogram measured at 10 mV/s. Bottom panel displays the linear sweep voltammogram for the same systems at a scan rate of 100 mV/s. The dashed lines show the intersection of the rising current density (ID) with the ID = 0 mA/cm^2^ representing the oxidation onset potential. The change in current density over a varied potential range is plotted for the oxidative forward scan of the first trace for each composition.

Gouy–Chapman theory predicts that such molecular arrangement should result in a U-shaped capacitance.^54,55^ Indeed, the experimental capacitance curves (Figure2) show such behavior, corroborating the correct description of the interface derived from the MD simulation. However, the experiments also reveal that diglyme/NaTFSI capacitance profile differs significantly from those of the other two systems, which is probably a consequence of the different ionic speciation observed for each system. The difference in the experimental profiles as compared to the theory is likely from the assumption that contact ion pairs contribute similarly to the Poisson potential as free ions. The difference in ionic speciation giving rise to the observe experimental behavior is discussed in detail, in the proceeding sections, in terms of the variations in sodium solvation environments at the interface.

**Figure 3:**
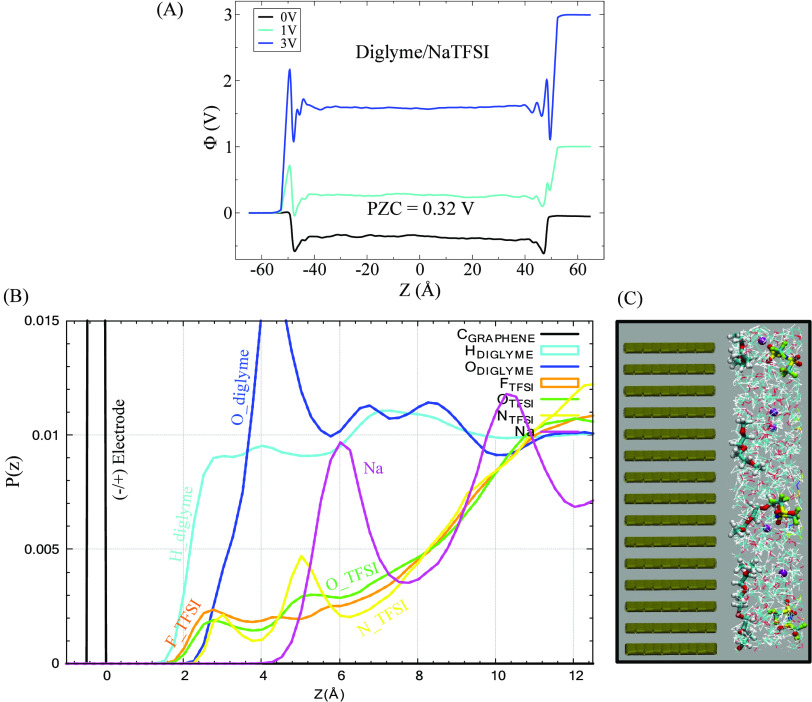
(a) Poisson potentials across the diglyme/NaTFSI simulation cell at applied potential differences of 0 V (black), 1 V (cyan), and 3 V (blue). The value for the potential of zero charge (PZC) is also provided. (b) Normalized probability density distribution (normalized to have unit area) of each atom in NaTFSI/diglyme system at zero applied voltage. (c) Snapshot from the simulation of NaTFSI/diglyme at 0 V showing the interfacial layer atoms that are 12 Å away from the electrode (H, O, C, N, F, S, Na atoms, and electrode in the snapshot are represented in white, red, cyan, blue, fluorine, yellow, purple, and tan, respectively).

The potential of zero charge (PZC)^56^ offers an estimation of the amount of work needed to carry the electrolyte ions to the vicinity of the electrode surface. The observed potential of zero charge is attributed to a higher concentration of charged species at the interfaces. For example, the accumulation of anions featuring electronegative elements like oxygen near the electrode surface at 0V results in a negative PZC.^57^ In this study, the observed PZC values were found to be positive: 0.32 V for dyglime/NaTFSI, 0.45 V for triglyme/NaTFSI, and 0.48 V for tetraglyme/NaTFSI. These values are in a range of 200 mV in agreement with the experimental observations. Note that the values of the PZC cannot be directly compared between experiment and theory since the reference states are different in the two cases, where simulations typically recover the PZC values in terms of the absolute electrode potential.^58^ When examining the atomic distributions of each species near the electrode surface of the diglyme/NaTFSI system held at zero applied potential, it is observed that the hydrogen atoms of diglyme stay closest to the electrode and that the weakly charged fluorine atoms of TFSI molecules are located next to hydrogens, away from the surface, followed by oxygens of TFSI and diglyme. Figure 3b shows the normalized probability density distribution for each atomic species near the electrode surface of the diglyme/NaTFSI system at 0 V, and Figure 3c provides a snapshot taken from the simulation depicting this arrangement of interfacial atoms at the same potential.

The positive PZC values observed in glyme/NaTFSI systems can be attributed to the greater abundance of hydrogen atoms near the surface relative to the number of electronegative species and fluorine and oxygen atoms in the TFSI anions. Moreover, the PZC value rises from diglyme to tetraglyme, indicating that the PZC value increases with the increase in glyme-chain length since more hydrogens contribute more to the positive value. However, the increment in the value from triglyme to tetraglyme is relatively modest compared to the increase observed from diglyme to triglyme. This effect is attributed to the high concentration of TFSI anions at the uncharged electrode surface (at 0V) from diglyme to tetraglyme.

### Comparison of the Differences in the Sodium Solvation Environments at the Interfacial Regions

**Figure 4:**
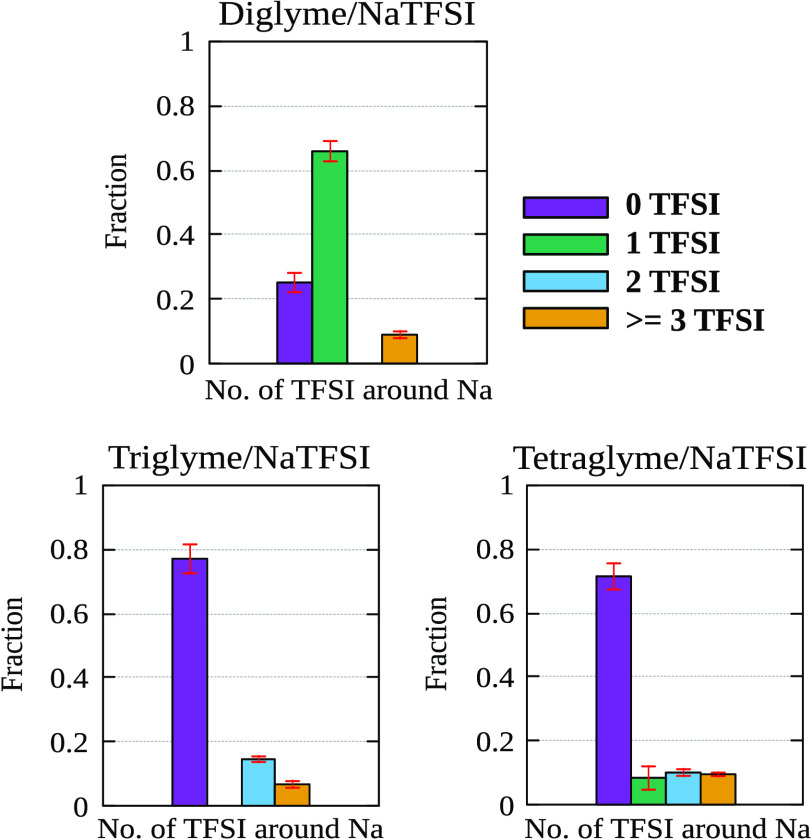
Fractional distribution of TFSI anions in the first solvation shell of sodium in bulk simulations (without the presence of electrodes) of (a) diglyme/NaTFSI, (b) triglyme/NaTFSI, and (c) tetraglyme/NaTFSI systems.

The distribution of the number of TFSI ions coordinated with sodium ions were calculated from the bulk simulations (without the presence of electrodes) for each glyme/NaTFSI system using a Na-N(TFSI) based distance definition. Figure 4 shows the populations calculated from bulk simulations of each system where purple, green, blue, and yellow stand for 0, 1, 2, 3, or more TFSI molecules coordinated to a sodium ion. The results obtained for bulk systems, for the fractions of TFSI in the first solvation shell of Na^+^, are in good agreement with those reported previously using the same modified PCFF.^46^ The key observation here is the notable absence of Na^+^ coordination to 2 TFSI (in bulk simulations of diglyme/NaTFSI systems) compared to triglyme and tetraglyme. The highest population seen in diglyme corresponds to Na^+^ coordination with 1 TFSI followed by the Na^+^ solvation structures with no TFSI present. In contrast, bulk triglyme/NaTFSI systems show a predominant fraction of sodium coordinating with 0 TFSI, followed by 2 TFSI and 3 or more TFSI. Moreover, the presence of 1 TFSI coordinated to Na^+^ is minimal. The tetraglyme/NaTFSI systems present populations for each class of solvation structure (0, 1, 2, and 3 or more TFSI coordinated to Na^+^) and are very similar to that of triglyme/NaTFSI.

**Figure 5:**
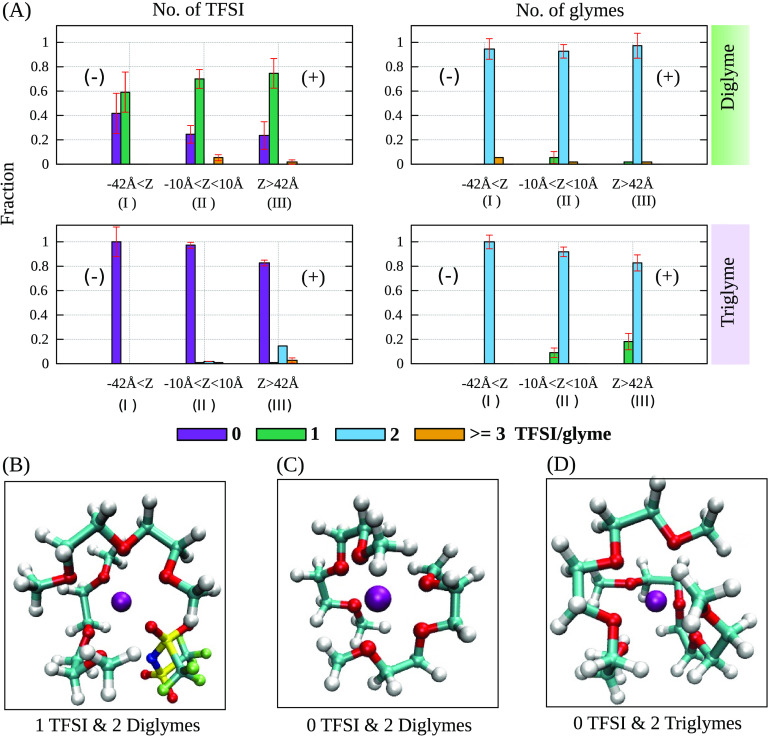
(a) Distribution of the fractions of TFSI anions (left panels) and glyme molecules (right panels) in the first solvation shell of sodium at 1V for diglyme/NaTFSI (top panels) and triglyme/NaTFSI (bottom panels). Inside each panel, three regions, I, II, and III, represent the fractional distribution of each TFSI and glymes at the negative interfacial region, bulk-like region, and positive interfacial region, respectively. Also, snapshots of the sodium solvation- structures present (b) 1 TFSI/ 2 diglymes (major), (c) 0 TFSI/ 2 diglymes, and (d) 0 TFSI/ 2 triglymes (major) are shown.

This solvation structure analysis in the cell shows three distinct regions: (I) negative interfacial region (−42 Å < *z*), (II) bulk-like region (−10 Å < *z* < 10 Å), and (III) positive interfacial region (42 Å > *z*) (see Figure 1a). Note that the cutoff values for interfacial regions considered here correspond to the Poisson potential drop along the *z*-axis near the interfaces (Figure 3a). Figure 5 presents both the TFSI and glyme fractions calculated for diglyme/NaTFSI and triglyme/NaTFSI at 1V. Figures S3–S5 in the SI compare these fractions as a function of voltage applied for all electrolytes.

It is noticed that across all glyme cases and across all of the regions, the major solvation structure of the sodium ion comprises two glymes, with the sole exception being the presence of 1 TFSI in the diglyme case. Furthermore, as the glyme chain length increases, TFSI is notably absent from these major sodium solvation structures. Also, when going from 0 to 3 V, the dominant sodium solvation structure in each region remains as the major sodium solvation structure for each system. Most importantly, there is no substantial deviation from the solvation structures observed in the bulk-like region (region II) when considering the influence of the polarized electrode on solvation structures found in interfacial regions (regions I and III). These results indicate that the solvation structures near the electrodes in the diglyme system exhibit clear distinctions from those observed in the proximity of the electrodes in the triglyme and tetraglyme systems. A more detailed description of the cases is depicted below.

For the zero voltage cases (0 V), an observation from all three glyme/NaTFSI systems is that once the electrolyte is confined between the electrodes, not all of the solvation structures observed in bulk simulations are presenting the same fractions. In diglyme/NaTFSI, only two distinct sodium solvation structures emerge: the primary structure entails 2 diglymes and 1 TFSI, with the secondary structure involving 2 diglymes and no TFSI, as shown in Figure 5. In the case of triglyme/NaTFSI, the structure having two triglyme molecules coordinating with a sodium cation stands out as the predominant configuration, while the structure involving 1 triglyme and 2 TFSI falls within the margin of error and is negligible (Figure 5). The distance of the isolated cation to the electrode in the absence of solvent was determined to be 1.86 Å (see Supporting Information for details). In the case of these electrode simulations, the distributions of Na^+^ starts at around 4 Å from the electrode and peaks at around 6 Å in all cases (see Figure 2 and Figure S1), as compared to 1.86 Å for the isolated cation case. Finally, in the tetraglyme/NaTFSI system, in contrast to the range of Na^+^ solvation structures observed in bulk, a single prominent sodium solvation configuration (2 tetraglyme with 0 TFSI as depicted in Figure S5, SI) prevails consistently across all of the cell regions.

For the nonzero voltage cases, across all glyme systems, there is an emergence or an increase in the fractions of minor structures featuring 2 or more TFSI in the sodium solvation structure in region III (at the right, positive electrode). In region I (the surface of the negative electrode) of the diglyme/NaTFSI system, the two solvation structures are still evident, and their fractional composition experiences minimal changes from 0 to 3 V. Conversely, region I has essentially only the major solvation structure in the triglyme (Figure S4) and tetraglyme (Figure S5) systems, which is two glymes and zero TFSI coordinating with sodium. This observation aligns with the density profile of TFSI, which displays a peak in the negative interfacial region at 3 V for diglyme systems but not for triglyme and tetraglyme systems (Figure S1 in the SI). In the latter two, with more negative potentials, TFSI is absent whereas it is present in diglyme systems. Within region III in the diglyme system, the primary sodium solvation structure remains unaltered at positive voltages, with only fractional shifts occurring in minor solvation structures (Figure S3 of the SI). One of these minor solvation structures emerges at the positive electrode, where 1 diglyme and 3 TFSI molecules coordinate with sodium and a negatively charged solvation structure due to excess of TFSI ions. At 3 V, on the contrary, the dominant sodium solvation structure diminishes notably for both triglyme and tetraglyme, with triglyme experiencing a significant reduction and tetraglyme showing a slight decrease (Figures S4 and S5, SI).

The lack of a marked influence of the electrodes on the solvation structures for all three systems may be due to the very low dielectric constant values of the glyme molecules. The dielectric constants of ether-based electrolytes are around 7, which are much lower than those of carbonate-based electrolytes. For example, ethylene carbonate (EC) has a dielectric constant of around 89, and propylene carbonate (PC) has around 64.^34^ Such electrolytes would experience a considerable influence from the electrode surface, likely leading to solvation environments completely different from that of the bulk-like region.

The validity of the ionic speciation at the electrolyte–electrode interface derived from the molecular dynamics simulations was evaluated by comparing the experimental electrochemical window of the electrolyte with the oxidation stability of prominent solvation structures in glyme/NaTFSI systems predicted by quantum chemical methods.

Further support for the agreement of simulation and experimental findings is obtained from the HOMO–LUMO energy differences from electronic structure calculations for the primary solvation structures (1 TFSI/2 diglyme, 0 TFSI/2 triglyme, and 0 TFSI/2 tetraglyme) for each glyme/NaTFSI system. This metric was used because it has been previously shown that the HOMO–LUMO gap correlates with electrochemical stability.^59^ It has also been previously demonstrated that other processes, such as proton transfer, can alter the observed electrochemical stability.^60^ However, the similarity of the electrolytes studied here allows us to use the HOMO–LUMO gap methodology, because it is likely that the presence of different oxidative pathways will be the same for the different glyme/NaTFSI systems.

Cyclic voltammetry conducted on three glyme/NaTFSI electrolyte systems (Figure 2) reveals that the oxidation of the glyme/NaTFSI electrolyte have the following trend: diglyme/NaTFSI < triglyme/NaTFSI < tetraglyme/NaTFSI. In particular, the oxidation potential of diglyme/NaTFSI is observed to be significantly lower than that of triglyme/NaTFSI and tetraglyme/NaTFSI systems. This observation aligns to the ionic characterization of the diglyme/NaTFSI system, which is clearly different from that seen in the triglyme/NaTFSI and tetraglyme/NaTFSI systems as previously described.

**Figure 6:**
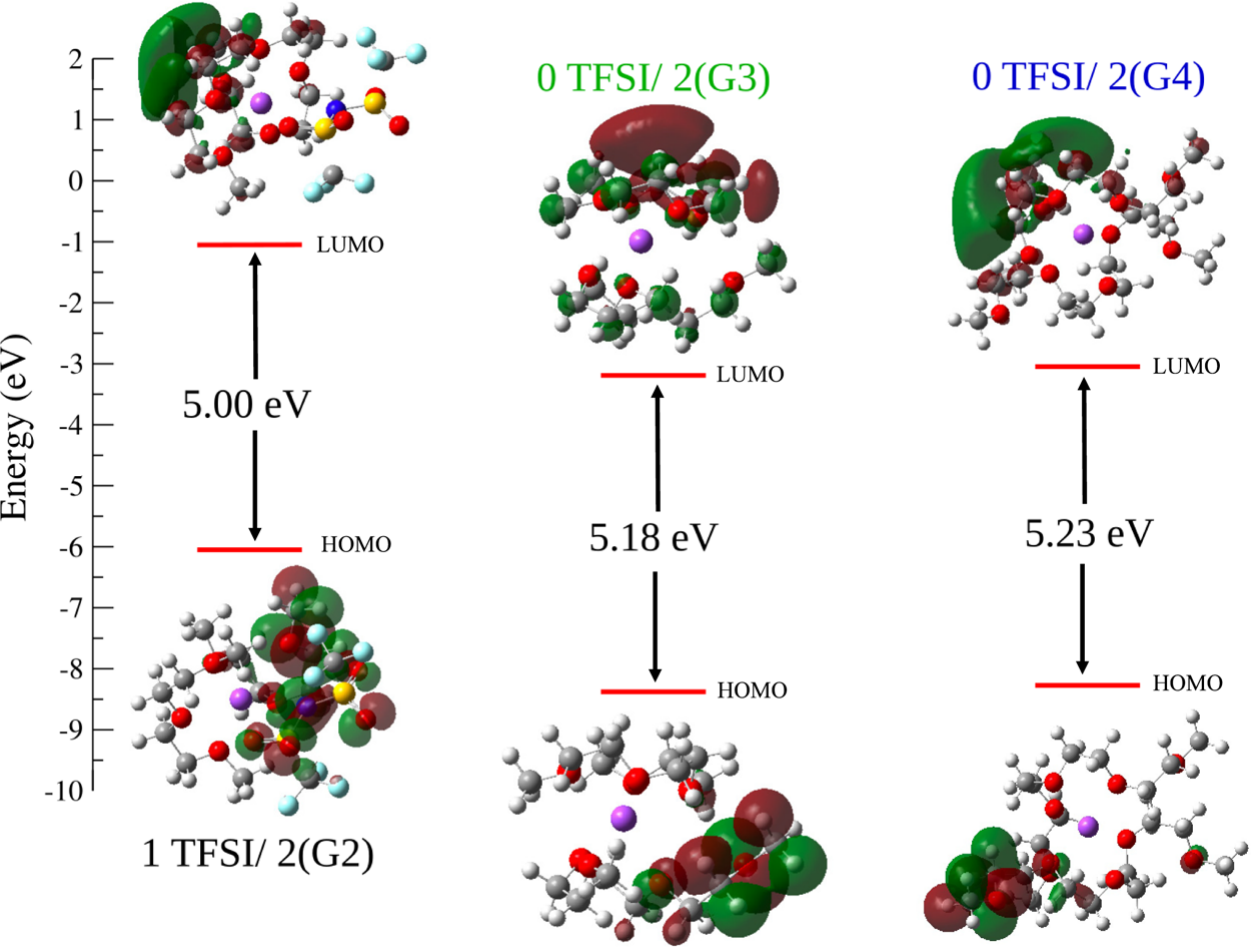
HOMO–LUMO energy difference obtained from electronic structure calculations at the PBE level of theory using 6–31+G(d,p) as the basis set for major solvation structures of diglyme (G2), triglyme (G3), and tetraglyme (G4) systems.

A more quantitative assessment of the experimental findings using the simulation results is obtained from the HOMO–LUMO energy differences from electronic structure calculations for the primary solvation structures (1 TFSI/2 diglyme, 0 TFSI/2 triglyme, and 0 TFSI/2 tetraglyme) for each glyme/NaTFSI system. The energy gap values for prominent solvation structures in each glyme/NaTFSI system (Figure 6) show an ascending order of 1 TFSI/2 diglyme < 0 TFSI/2 triglyme < 0 TFSI/2 tetraglyme. The increase in the energy gap has a direct correspondence to the experiment. However, the computational results also show a more pronounced increase in the energy gap from diglyme to triglyme than from triglyme to tetraglyme, explaining the experimental observations. In other words, the simulations suggest that the electrochemical window of the different glyme/NaTFSI system likely arises from the different chemical speciation at the interface. In particular, it is apparent that the change in the ionic speciation is responsible for the size of the electrochemical window observed in these systems.

Finally, it is worthwhile to highlight that the EDL features, evidenced in the Poisson potential plots (see Figure 3a and Figure S2), do not vary significantly as a function of the glyme length nor do solvation structures change in the interfacial regions, compared with the respective results for bulk simulations. For example, molecular simulations with experimental vibrational sum-frequency generation (vSFG) spectra of the interface between water and graphene oxide sheets have shown that water ordering at the interface significantly differs from bulk water due to hydrogen bonding interactions.^59,61^ In platinum/water layers, the behavior of water near the interface depends on the metal surface structure.^62^ Furthermore, molecular dynamics simulations conducted on an aqueous ionic solution near a model metallic wall held at a constant potential show a strong attraction and ordering of water molecules at the electrode surface.^63^ The dielectric constants of the ether solvents are significantly lower than that of water, and the chelation of the cations by the oxygen atoms of the ether (glyme) solvent is the driving force for the dissolution of these salts in solution. Hence, it is not surprising that chelation is not significantly affected by the presence of an electrode or by the applied voltage on the electrodes, and the formation of EDLs in these electrolytes depends almost exclusively on the speciation that is observed in each system, being more similar between the triglyme and tetraglyme cases and in keeping with the CV measurements.

## Conclusions

In this study, a molecular dynamics investigation is conducted to explore the molecular interactions occurring at the interface between sodium/glyme-based electrolytes and polarizable graphite electrodes under constant potential differences. This research encompasses the simulation of three systems involving 1 M NaTFSI/glyme-based electrolytes, specifically diglyme, triglyme, and tetraglyme, and carried out at three different applied voltages (0, 1, and 3 V) across the two electrodes. The interface’s solvation environments were compared against bulk solvation environments, specifically focusing on the impact of glyme length and applied voltage. The density profiles plotted along the *z*-axis in the interfacial regions of each system reveal that, even when subjected to the voltages used in this study, the ability to draw sodium ions closer to the electrode surface is unaffected, unlike the TFSI anions and glyme molecules. Instead, the sodium ions tend to maintain their solvation with glyme molecules at all potential differences examined. These simulations were intended to reproduce the main features of an electrolyte/electrode model at voltages relevant to battery operations. However, at much higher voltages, one would expect the deformation of the solvation structures.

The observed PZC values for the glyme/NaTFSI systems were found to be positive, primarily due to the proximity of glyme hydrogens to the electrode surface at zero applied voltage. Most importantly, the results of the study showcase the features at the interfacial layers of the diglyme/TFSI system compared to the triglyme/TFSI and tetraglyme/TFSI systems, where the latter two show quite similar characteristics. At 3 V, the negative interfacial layer of the diglyme/TFSI system consists of two solvation structures, with the primary structure consisting of 1 TFSI with two diglymes and a secondary structure with two glymes without TFSI. In contrast, the negative interfacial regions of triglyme and tetraglyme TFSI systems are dominated by one solvation structure, which consists of two glymes each with zero TFSI. At 3 V, in the positive interfacial region of the diglyme system, the primary sodium solvation structure remains unaltered, with only fractional shifts occurring in minor solvation structures. Conversely, for triglyme and tetraglyme, the fraction of major solvation structure changes at high positive potentials. The disparity between the diglyme/NaTFSI systems in contrast to the triglyme and tetraglyme NaTFSI systems was consistent with the results of cyclic voltammetry investigations and with the comparisons of HOMO–LUMO energy levels within the major solvation structures for each respective system. The analysis of the electrical double layer, obtained through Poisson potential calculations in the MD simulations, shows that the Na^+^/glyme is not sensitive to the applied voltages. These results, along with the calculated PZC values and experimental CV measurements, confirm that the triglyme and tetraglyme dynamics are similar with diglyme acting as the outsider solvent. The insights acquired on the solvation environments within the interfacial regions in this study contribute to our comprehension of the decomposition pathways influencing the creation of SEIs derived from different glyme/NaTFSI systems. Additionally, these findings will aid in future investigations of reactive processes such as charge transfer and sodium intercalation-deintercalation at the electrode–electrolyte interface.

## Data Availability

The data that support the findings of this study are available from the corresponding author upon reasonable request.

## Supporting Information Available

Graphs showing the normalized distribution of each species as a function of distance from the electrodes for different applied voltages, the Poisson potential across the cell for each system at different applied voltages, and the fraction of different solvation environments for the different glymes as a function of applied voltages; force field parameters in the LAMMPS input file format (PDF)
Input structures for the static quantum calculations (ZIP)
