# Supplementary material for: Probing the Electrode–Electrolyte Interface of Sodium/Glyme-Based Battery Electrolytes
Source: J Phys Chem C Nanomater Interfaces. 2024 Mar 27;128(14):5798–808. doi: 10.1021/acs.jpcc.3c08083 (PMC11017320; doi:10.1021/acs.jpcc.3c08083)
Supplement: Supplementary file 1 — jp3c08083_si_001.pdf [file jp3c08083_si_001.pdf]

Supporting Information
Probing the Electrode-Electrolyte Interface of Sodium/Glyme-Based Battery Electrolytes

Dodangodage Ishara Senadheera,[1§] Orlando Carrillo-Bohorquez,[1§] Ernest O. Nachaki,[1] Ryan Jorn,[2] Daniel Kuroda[1,*] and Revati Kumar[1,*]

[1]Department of Chemistry, 232 Choppin Hall, Louisiana State University, Baton Rouge, Louisiana 70803, USA

[2]Department of Chemistry, Villanova University, Villanova, PA 19085, USA

[*]*email:* revatik@lsu.edu; dkuroda@lsu.edu

[§]contributed equally

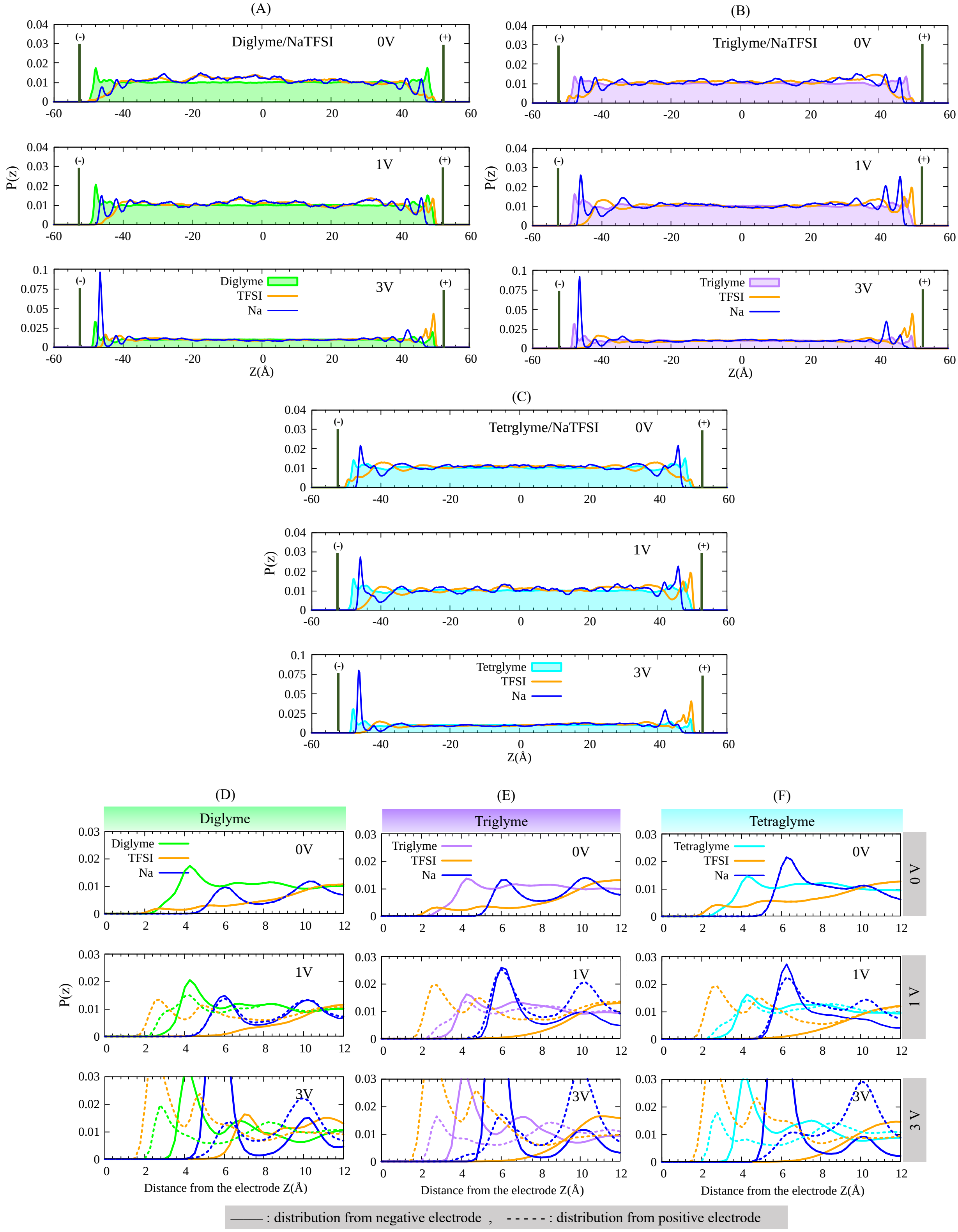

Figure S1. Normalized probability density distributions of each species (considering the oxygen atoms of glymes and TFSI) across the cell as a function of *z* in (a) diglyme/NaTFSI , (b)

triglyme/NaTFSI, (c) tetraglyme/NaTFSI systems and compared at three applied potential differences, 0V, 1V, 3V (shown top to down in panels in each window, respectively). Comparison of the distributions near the electrode surfaces for (d) diglyme/NaTFSI, (e) triglyme/NaTFSI, and (f) tetraglyme/NaTFSI (solid lines represent the distribution from the negative electrode and dashed lines represent the distribution from the positive electrode and $TFSI^-$, Na^+, diglyme, triglyme, and tetraglyme are represented by orange, blue, green, purple, and cyan lines, respectively). The positions of the electrode surfaces in figures a, b and c are depicted by vertical lines.

Comparison of the normalized density profiles along the z-axis at the interfacial regions

Figure S1d-f provides a complete comparison of the salient features of the interfacial layers forming at the negative electrode and positive electrode surfaces as a function of increasing glyme-chain length (from left to right) and as a function of applied potential (panels showing top to bottom). In all panels of Figure S1d-f, the solid lines represent the normalized density distribution from the negative electrode, while dashed lines represent the distribution from the positive one. The first interfacial layers at 0V (top panels in Figures S1d-f) span between 1.6 and 5.6 Å from the electrode surfaces, with a first broad peak of TFSI oxygen atoms appearing around 2.8 Å and a clear first peak of glyme oxygen atoms around 4.4 Å. A second interfacial layer is between 4 and 7.2 Å from the electrode surfaces, with the first peak of Na^+ showing up at 6 Å. When examining from left to right, the peak heights of TFSI anions and Na^+ ions at 0V show an increase from diglyme to tetraglyme. At 1V (middle panels in Figures S1d-f), the first peak positions remain the same for Na^+ ions at both positive and negative electrodes, with a slight increase in peak height for the diglyme/TFSI system and considerable changes in the Na^+ peak heights for the triglyme/TFSI and tetraglyme/NaTFSI systems at both interfaces. The glyme distributions at 1V indicate that even though the peak positions representing glymes remain the same at the negative electrode (solid lines), a shoulder appears around 2.8 Å from the positive electrode (dashed lines), which is an indication of glyme oxygens tendency to compete with TFSI oxygen atoms to get closer to the positively charged surface. The distribution for TFSI oxygen atoms at 1V shows clear

differences between the two interfaces. While the peak height for TFSI appearing at 2.8 Å away from the positive electrode increases (orange dashed lines), the peak at the negative electrode depletes significantly (orange solid lines).

At higher voltage differences (3V, bottom panels in Figures S1d-f), the TFSI anion distributions at the negative electrode show distinct features from diglyme to tetraglyme. Most importantly, the distribution of the TFSI oxygens at the negative electrode (solid lines) for diglyme/NaTFSI system shows a peak at around 7 Å away from the surface, while there is no such a peak appearing for triglyme and tetraglyme at positive potentials. Apart from this significant observation, in all three electrolytes the peak positions remain the same for all the species. The true peak heights of the distributions at interfacial regions for the 3V case can be seen in Figures S1a-c.

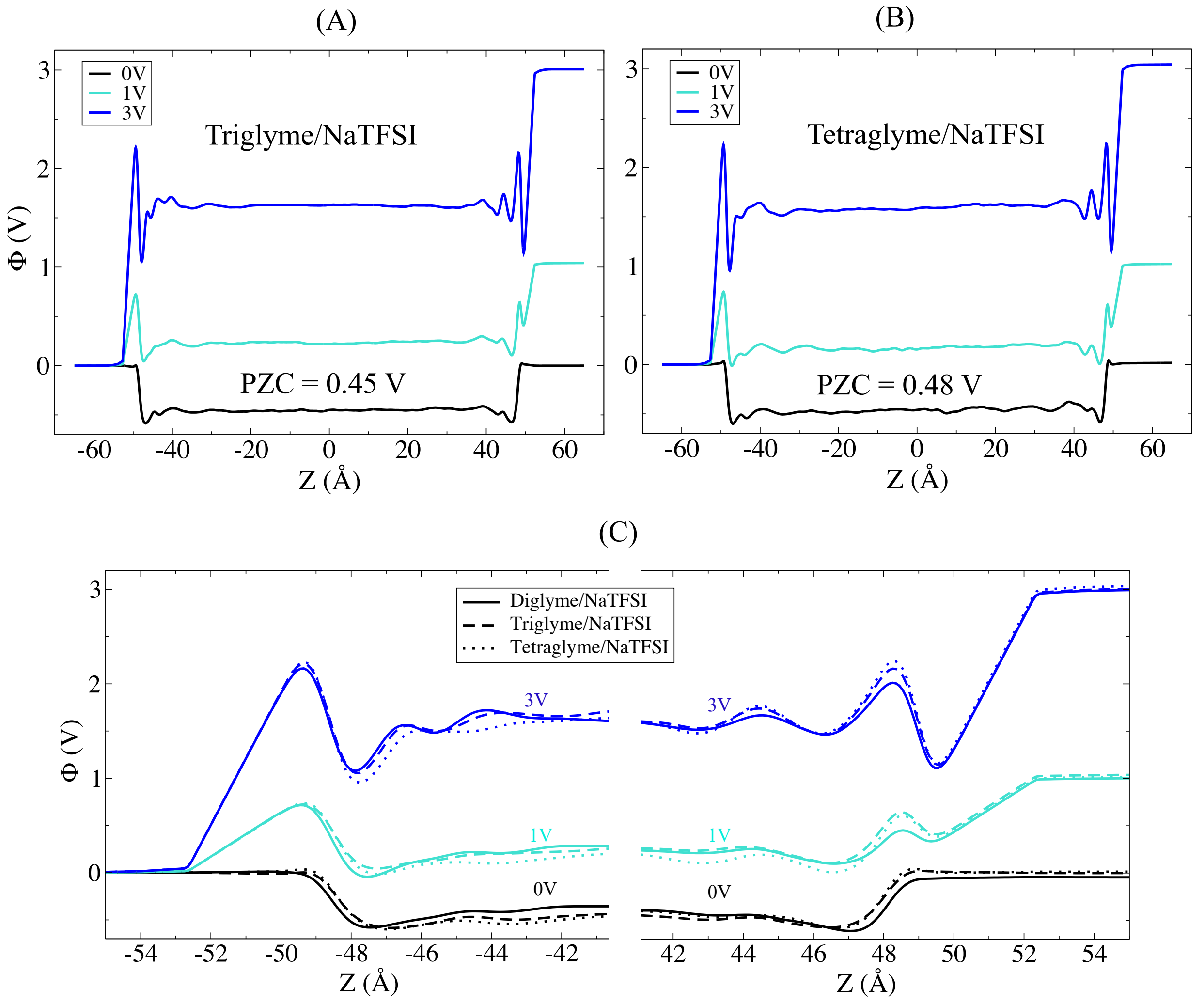

Figure S2. Poisson potentials across (a) the triglyme/NaTFSI and (b) the tetraglyme/NaTFSI simulation cell at applied potentials differences of 0V (black), 1V (cyan), and 3V (blue). (c) Zoomed-in view of the Poisson profile at the interfacial regions for diglyme/NaTFSI (solid lines), triglyme/NaTFSI (dashed lines), and tetraglyme/NaTFSI (dotted lines).

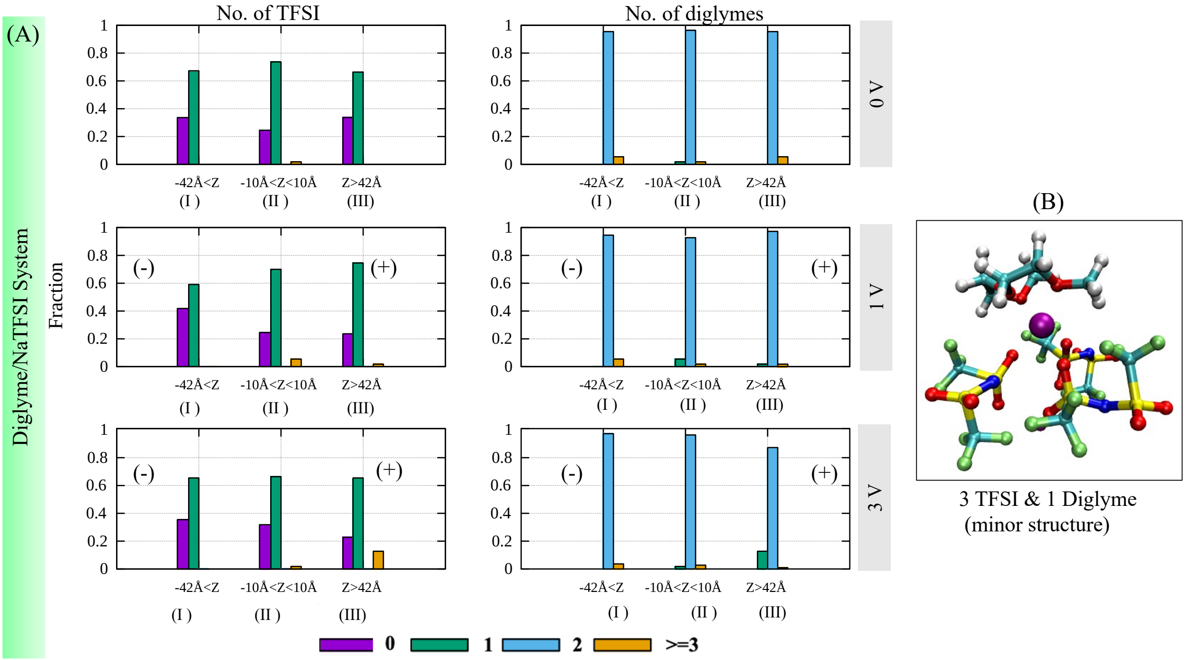

Figure S3. (a) Distribution of the fractions of TFSI anions (left panels) and diglyme molecules (right panels) in the first solvation shell of sodium as a function of applied voltages (panels showing top to bottom) for the diglyme/NaTFSI system. Inside each panel, three regions, I, II, and III, represent the fractional distribution of each TFSI and diglyme at the negative interfacial region, bulklike region, and positive interfacial region, respectively. (b) Also, a snapshot of the minor sodium solvation structure presents in the diglyme/NaTFSI system, 3 TFSI/ 1 diglyme is shown.

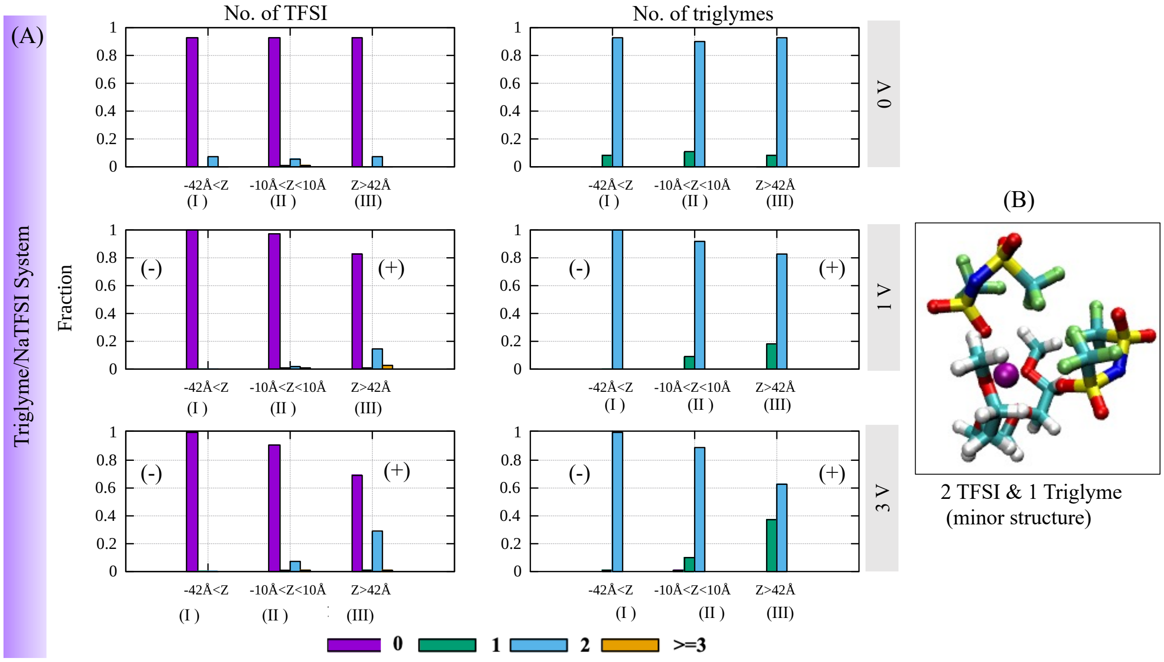

Figure S4. (a) Distribution of the fractions of TFSI anions (left panels) and triglyme molecules (right panels) in the first solvation shell of sodium as a function of applied voltages (panels showing top to bottom) for the triglyme/NaTFSI system. (b) A snapshot of the minor sodium solvation structure presents in the triglyme/NaTFSI system, 2 TFSI/ 1 triglyme.

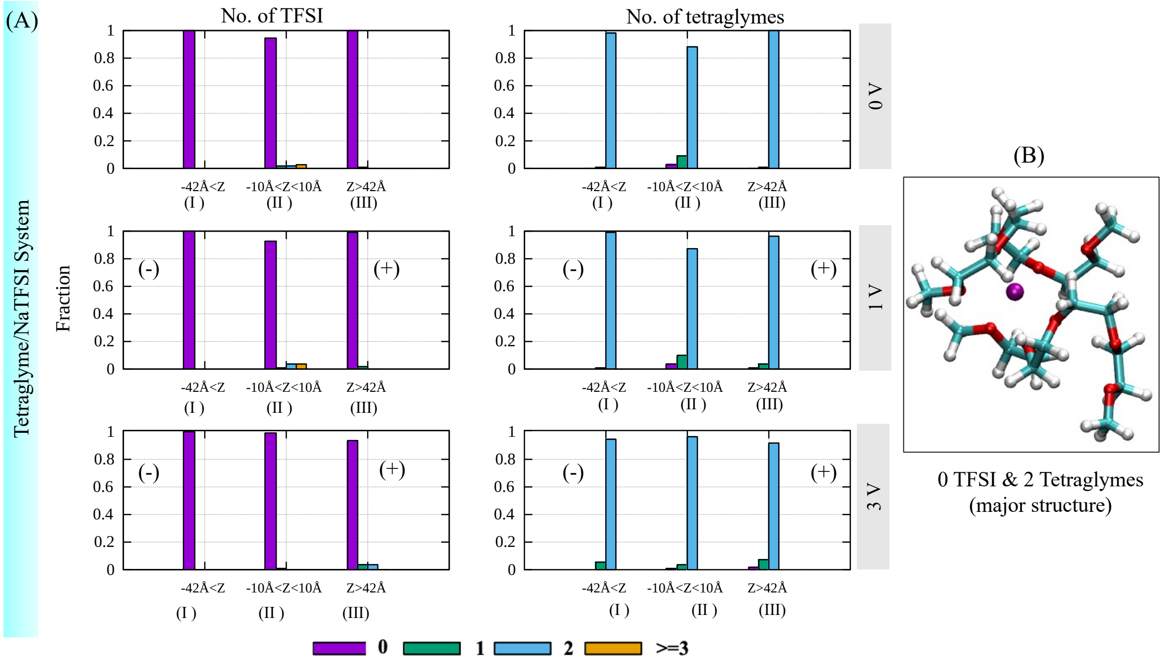

Figure S5. (a) Distribution of the fractions of TFSI anions (left panels) and tetraglyme molecules (right panels) in the first solvation shell of sodium as a function of applied voltages (panels showing top to bottom) for the tetraglyme/NaTFSI system. (b) A snapshot of the major sodium solvation structure present in the tetraglyme/NaTFSI system, 0 TFSI/ 2 tetraglyme.

Table S1. Number of molecules of each species in the glyme/NaTFSI systems under study, along with the final volume occupied by every electrolyte. For the electrode (CPM) calculations, simulation boxes have dimensions of (50.20 × 50.30 × 129.6) $Å^3$, and that of the electrode is (50.20 × 50.30 × 12.28) $Å^3$, and the inter-electrode space is 105.0 Å. Bulk-like equilibrated simulations were used as starting points for the CPM calculations, and molecules or ions were removed/added in each case to fill up properly the space between electrodes while keeping the correct molarity (1 M).

System	NaTFSI	Glymes	Final Electrolyte Volume
Diglyme/NaTFSI (bulk)	150	895	(50.38 × 50.38 × 100.8) $Å^3$
Triglyme/NaTFSI (bulk)	150	628	(49.77 × 49.77 × 99.54) $Å^3$
Tetraglyme/NaTFSI (bulk)	150	590	(50.33 × 50.33 × 100.7) $Å^3$
Diglyme/NaTFSI (electrode)	148	867	(50.20 × 50.30 × 101.1) $Å^3$
Triglyme/NaTFSI (electrode)-I	151	661	(50.20 × 50.30 × 100.9) $Å^3$
Triglyme/NaTFSI (electrode)-II	151	665	(50.20 × 50.30 × 101.0) $Å^3$
Tetraglyme/NaTFSI (electrode)-I	150	558	(50.20 × 50.30 × 100.8) $Å^3$
Tetraglyme/NaTFSI (electrode)-II	150	552	(50.20 × 50.30 × 101.1) $Å^3$

Cation adsorption distance: A simulation of an isolated Na^+ cation at 300 K between the two electrodes at 0V for was run for 1 ns with a timestep of 1 fs in the same simulation box as described in Table S1 for CPM simulations (NVT ensemble). On average the closest distance of the Na+ from an electrode was found to be 1.88 Å This value agrees well with the adsorption distance calculated through the sixth power combination rule between carbon and Na^+ (1.86 Å).

HOMO-LUMO calculation for 0 TFSI/2(G2): The energy gap for 0 TFSI/2(G2) was found to be 5.63 eV in this work.

Partial charges of the TFSI ion (From J. Chemical Physics, 2021 154, 184505):

C: 0.582137
F: -0.219159
S: 1.136055
O: -0.602367
N: -0.712098

LAMMPS input parameters:

Below, the settings for the NaTFSI in glyme simulations are presented, along with the atoms type utilized.

<u>*Glyme atoms type:*</u>

1 O

2 O
3 C
4 C
5 C
6 H
7 H
8 H

<u>NaTFSI *atoms type:*</u>

9 S
10 O
11 C
12 F
13 N
14 Na

bond_style hybrid class2 harmonic

```
bond_coeff    1  class2    1.1010 341.0000 -691.8900  844.6000
bond_coeff    2  class2    1.4200 400.3954 -835.1951 1313.0142
bond_coeff    3  class2    1.5330 299.6700 -501.7700  679.8100
bond_coeff    4  harmonic 441.80 1.323
bond_coeff    5  harmonic 235.42 1.818
bond_coeff    6  harmonic 637.07 1.442
bond_coeff    7  harmonic 372.01 1.570
```

angle_style hybrid class2 harmonic

```
angle_coeff   1   class2       107.6600 39.6410   -12.9210   -2.4318
angle_coeff   1   class2 bb      5.3316   1.1010     1.1010
angle_coeff   1   class2 ba     18.1030 18.1030      1.1010    1.1010
angle_coeff   2   class2       108.7280 58.5446   -10.8088  -12.4006
angle_coeff   2   class2 bb     23.1979  1.1010      1.4200
angle_coeff   2   class2 ba      4.6189 55.3270      1.1010    1.4200
angle_coeff   3   class2       104.5000 35.7454   -10.0067   -6.2729
angle_coeff   3   class2 bb     -7.1131   1.4200     1.4200
angle_coeff   3   class2 ba     -2.8112  -2.8112     1.4200    1.4200
angle_coeff   4   class2       110.7700 41.4530   -10.6040    5.1290
angle_coeff   4   class2 bb      3.3872   1.5330     1.1010
angle_coeff   4   class2 ba     20.7540 11.4210      1.5330    1.1010
angle_coeff   5   class2       111.2700 54.5381    -8.3642  -13.0838
angle_coeff   5   class2 bb     11.4318  1.5330      1.4200
angle_coeff   5   class2 ba      2.6868 20.4033      1.5330    1.4200
angle_coeff   6   harmonic       93.332    107.1
```

```
angle_coeff   7   harmonic      82.935   111.8
angle_coeff   8   harmonic     103.967   102.6
angle_coeff   9   harmonic     115.798   118.5
angle_coeff   10  harmonic      94.288   113.6
angle_coeff   11  harmonic      97.514   100.2
angle_coeff   12  harmonic      80.186   125.6

dihedral_style  class2

dihedral_coeff 1        0.5302  0.0000  0.0000  0.0000 -0.2836  0.0000
dihedral_coeff 1 mbt   -6.8007 -4.6546 -1.4101  1.4200
dihedral_coeff 1 ebt   -0.6054  1.3339  0.9648 -0.1620  0.1564 -1.1408    1.1010    1.4200
dihedral_coeff 1 at    -1.8234  1.6393  0.5144 -0.7777  0.4340 -0.6653  108.7280  104.5000
dihedral_coeff 1 aat  -16.4438 108.728  104.5000
dihedral_coeff 1 bb13  0.0000  0.0000  0.0000

dihedral_coeff 2       -0.1820  0.0000 -0.1084  0.0000 -0.7047  0.0000
dihedral_coeff 2 mbt  -17.2585 -3.6157 -0.8364  1.5330
dihedral_coeff 2 ebt    1.0165  0.7553 -0.4609  1.0165  0.7553 -0.4609    1.4200     1.4200
dihedral_coeff 2 at     0.5511  0.9737 -0.6673  0.5511  0.9737  -0.6673 111.2700   111.2700
dihedral_coeff 2 aat  -14.0484 111.2700 111.2700
dihedral_coeff 2 bb13  0.0000  0.0000  0.0000

dihedral_coeff 3       -0.1435  0.0000  0.2530  0.0000 -0.0905  0.0000
dihedral_coeff 3 mbt -16.7975 -1.2296 -0.2750  1.5330
dihedral_coeff 3 ebt    0.9681  0.9551  0.0436  0.5903  0.6669  0.8584    1.1010     1.4200
dihedral_coeff 3 at     2.3668  2.4920 -1.0122 -0.1892  0.4918  0.7273  110.7700   111.2700
dihedral_coeff 3 aat  -20.2006 110.7700 111.2700
dihedral_coeff 3 bb13  0.0000  0.0000  0.0000

dihedral_coeff 4      -0.5203  0.0000 -0.3028  0.0000 -0.3450  0.0000
dihedral_coeff 4 mbt  -5.9288 -2.7007 -0.3175  1.4200
dihedral_coeff 4 ebt   -0.2456  1.0517 -0.7795  0.4741  1.2635  0.5576    1.5330     1.4200
dihedral_coeff 4 at    -2.7466  1.4877  -0.8955 0.5676  0.9450  0.0703  111.2700   104.5000
dihedral_coeff 4 aat  -19.0059 111.2700 104.5000
dihedral_coeff 4 bb13  0.0000  0.0000  0.0000

dihedral_coeff 5       -0.2432  0.0000  0.0617  0.0000 -0.1383  0.0000
dihedral_coeff 5 mbt  -14.2610 -0.5322 -0.4864  1.5330
dihedral_coeff 5 ebt    0.2130  0.3120  0.0777  0.2130  0.3120  0.0777    1.1010     1.1010
dihedral_coeff 5 at    -0.8085  0.5569 -0.2466 -0.8085  0.5569 -0.2466  110.7700   110.7700
dihedral_coeff 5 aat   -12.5640 110.7700 110.7700
dihedral_coeff 5 bb13  0.0000  0.0000  0.0000

dihedral_coeff 6        0 0 0 0 0.173 180
dihedral_coeff 6 mbt   0.0 0.0 0.0 0.0
```

```
dihedral_coeff 6 ebt    0.0 0.0 0.0 0.0 0.0 0.0 0.0 0.0
dihedral_coeff 6 at     0.0 0.0 0.0 0.0 0.0 0.0 0.0 0.0
dihedral_coeff 6 aat    0.0 0.0 0.0
dihedral_coeff 6 bb13  0.0 0.0 0.0

dihedral_coeff 7        0 0 0 0 -0.00179 180
dihedral_coeff 7 mbt   0.0 0.0 0.0 0.0
dihedral_coeff 7 ebt    0.0 0.0 0.0 0.0 0.0 0.0 0.0 0.0
dihedral_coeff 7 at     0.0 0.0 0.0 0.0 0.0 0.0 0.0 0.0
dihedral_coeff 7 aat    0.0 0.0 0.0
dihedral_coeff 7 bb13  0.0 0.0 0.0

dihedral_coeff 8        0 0 0 0 0.158 180
dihedral_coeff 8 mbt   0.0 0.0 0.0 0.0
dihedral_coeff 8 ebt    0.0 0.0 0.0 0.0 0.0 0.0 0.0 0.0
dihedral_coeff 8 at     0.0 0.0 0.0 0.0 0.0 0.0 0.0 0.0
dihedral_coeff 8 aat    0.0 0.0 0.0
dihedral_coeff 8 bb13  0.0 0.0 0.0

dihedral_coeff 9        3.916 180 -1.245 0 -0.382 180
dihedral_coeff 9 mbt   0.0 0.0 0.0 0.0
dihedral_coeff 9 ebt    0.0 0.0 0.0 0.0 0.0 0.0 0.0 0.0
dihedral_coeff 9 at     0.0 0.0 0.0 0.0 0.0 0.0 0.0 0.0
dihedral_coeff 9 aat    0.0 0.0 0.0
dihedral_coeff 9 bb13  0.0 0.0 0.0

improper_style class2

improper_coeff 1       0.0 0.0
improper_coeff 1 aa    2.4259  2.4259  2.1283 107.6600 108.7280 108.7280
improper_coeff 2       0.0 0.0
improper_coeff 2 aa   -0.3157  -0.3157 -0.3157 107.6600 107.6600 107.6600
improper_coeff 3       0.0 0.0
improper_coeff 3 aa    2.1283  2.4259  2.4259  108.7280  107.6600  108.7280
improper_coeff 4       0.0 0.0
improper_coeff 4 aa    3.9177  0.1689  2.5926  108.7280  111.2700  110.7700
improper_coeff 5       0.0 0.0
improper_coeff 5 aa    2.4259  2.1283  2.4259  108.7280  108.7280  107.6600
improper_coeff 6       0.0 0.0
improper_coeff 6 aa   -0.4825  0.2738  0.2738  110.7700  107.6600  110.7700
improper_coeff 7       0.0 0.0
improper_coeff 7 aa    3.9177  2.5926  0.1689  110.7700  111.2700  108.7280
improper_coeff 8       0.0 0.0
improper_coeff 8 aa    0.2738  -0.4825  0.2738  110.7700  110.7700  107.6600

pair_style lj/class2/coul/long 6.0 6.0
```

```
pair_modify mix sixthpower

pair_coeff 1  1   0.2400 3.5350
pair_coeff 2  2   0.2400 3.5350
pair_coeff 1  2   0.2400 3.5350
pair_coeff 3  3   0.0540 4.0100
pair_coeff 4  4   0.0540 4.0100
pair_coeff 5  5   0.0540 4.0100
pair_coeff 3  4   0.0540 4.0100
pair_coeff 4  5   0.0540 4.0100
pair_coeff 3  5   0.0540 4.0100
pair_coeff 6  6   0.0200 2.9950
pair_coeff 7  7   0.0200 2.9950
pair_coeff 8  8   0.0200 2.9950
pair_coeff 6  7   0.0200 2.9950
pair_coeff 7  8   0.0200 2.9950
pair_coeff 6  8   0.0200 2.9950

pair_coeff 9   9  0.3923 3.564
pair_coeff 10 10  0.3121 2.913
pair_coeff 11 11  0.2530 3.5809
pair_coeff 12 12  0.3533 2.6454
pair_coeff 13 13  0.106  4.07
pair_coeff 14 14  0.1281 3.2335
pair_coeff 1  14  0.1631 3.1651
```
